# The Immunomodulatory Properties of Vitamin D

**DOI:** 10.31138/mjr.33.1.7

**Published:** 2022-03-31

**Authors:** Lambros Athanassiou, Clio P. Mavragani, Michael Koutsilieris

**Affiliations:** Department of Physiology, Medical School, University of Athens, Athens, Greece

**Keywords:** vitamin D, immunomodulation, innate immunity, adaptive immunity, immune tolerance, vitamin D deficiency

## Abstract

Since its discovery, vitamin D was shown to have both immunostimulatory and immunomodulatory effects on the immune system. A growing body of evidence so far linked vitamin D deficiency with the development and severity of several systemic and organ specific autoimmune/inflammatory diseases, such as systemic lupus erythematosus, rheumatoid arthritis, inflammatory bowel disease, and multiple sclerosis. In the present report, the multiple and diverse effects of vitamin D on the immune system are reviewed.

## INTRODUCTION

While vitamin D is well-known for its actions on bone and mineral metabolism,^1,2^ extraskeletal effects are increasingly recognized^3,4^; its influences on the immune system have been the focus of intense research.^5–7^ In earlier years, immunostimulatory effects were recognised,^8^ followed by subsequent observations revealing the relationship of vitamin D deficiency^9,10^ with the development of autoimmune diseases,^5,10^ given the ability of vitamin D to induce immune tolerance.^11,12^ In rheumatoid arthritis, vitamin D deficiency has been found to be prevalent in patients with rheumatoid arthritis^13–16^ and inflammatory bowel disease^17^ in association with increased disease activity.^14,16^ Similar observations were made in patients with systemic lupus erythematosus^18–20^ and systemic sclerosis,^21^ with the reported associations with disease activity being rather conflicting.^18,22–24^ Vitamin D deficiency has been also observed in patients with multiple sclerosis (MS),^25–28^ and vitamin D administration may be a complementary agent in MS treatment.^26^

Vitamin D deficiency has also been reported in patients with diabetes mellitus type 1^29–32^ and has been implicated in the development of the disease,^30,33^ potentially through modulating inflammatory pathways.^34^ Vitamin D receptors have been found in many cells of the immune system,^35–38^ such as T lymphocytes^36,39,40^ and macrophages,^41^ among others. Moreover, 1a-hydroxylase, the enzyme responsible for the formation of the active compound of the vitamin D system, namely 1,25(OH)_2_D_3_, has been found to be expressed in cells of the immune system,^42–44^ thus enabling the formation and action of the active compound of the vitamin D system, namely 1,25(OH)_2_D_3_. Type I interferons (IFNs) (IFN α/β) are proteins that normally provide protection from viral infections, through induction of hundreds of genes implicated in antiviral response; the so-called “IFN signature”. A significant role of the type I interferon (IFN) system in the pathogenesis of systemic autoimmune diseases has been well documented.^45,46^ Vitamin D has been shown in an experimental lupus model to modulate interferon-1 responses.^47^ In the current review, the immunomodulatory properties of vitamin D are reviewed.

## VITAMIN D AND IMMUNITY

**Figure 1.**
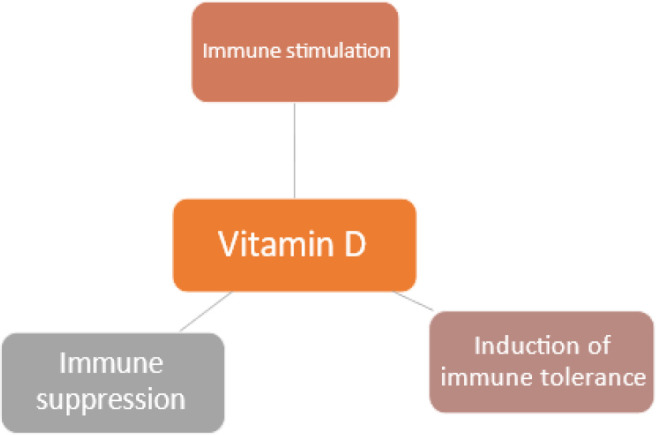
The effects of vitamin D on the immune system.^5,11,25,35^

**Figure 2.**
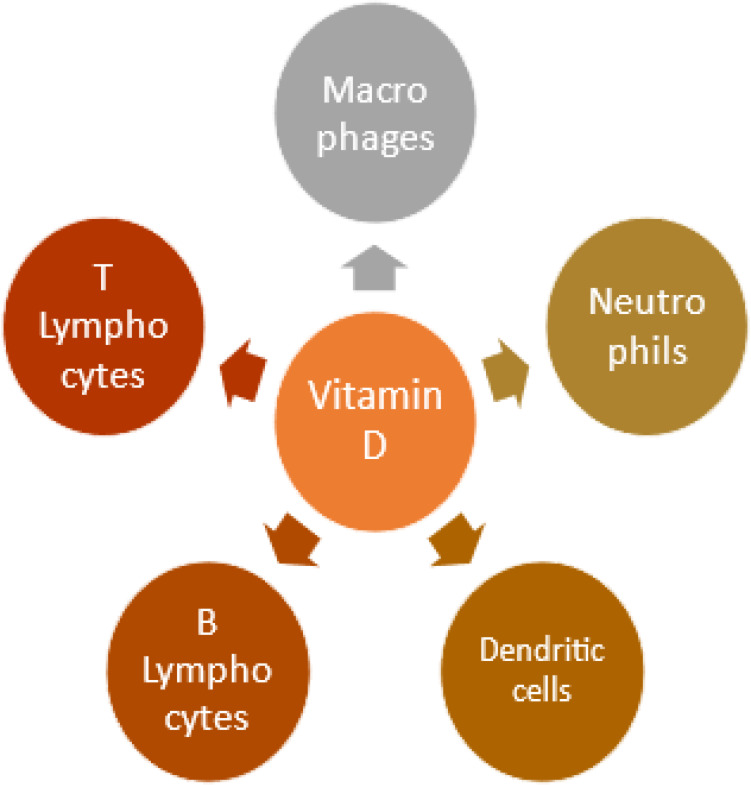
Cells of the immune system which are targets of vitamin D, macrophages,^41,59,61,79^ neutrophils,^38^ T lymphocytes,^39,40,87^ dendritic cells,^83^ B lymphocytes.^111^

While it is well established that vitamin D enhances intestinal calcium absorption, an effect mediated via regulation of calcium transport proteins in the small intestine,^48^ exhibiting a central role in the maintenance of bone health, extra skeletal actions are less explored. Amongst them extremely important are its effects on the immune system (**Figure 1**). Cells of the immune system harbour the vitamin D activating enzyme 1-α-hydroxylase and express the vitamin D receptor (VDR).^43,44^ The extra-renal 1-α-hydroxylase is not regulated by PTH and thus production of 1,25(OH)_2_D_3_ is dependent on concentrations of the substrate 25(OH)D_3_ and it may be regulated by inflammatory signals, such as lipopolysaccharide and cytokines.^42,49^ Cells of the immune system which express the VDR and harbour 1-α-hydroxylase are macrophages, T cells, dendritic cells, monocytes, and B cells^36,50^ (**Figure 2**).

Vitamin D is involved both in the regulation of the innate immunity as it enhances the body defence system against microbes and other pathogenic organisms, as well as in the modulation of the adaptive immune system through direct effects on T cell activation and on the phenotype and function of antigen-presenting cells; in particular, dendritic cells.

## VITAMIN D AND THE INNATE IMMUNE SYSTEM

Vitamin D regulates the innate immune system.^2,5,51^ The innate immune system -an older evolutionary defence strategy- is a first line of defence against infection,^52,53^ and one of the two main immunity arms in vertebrates, including humans.^53^ Its major functions include recruitment of immune cells, activation of the complement cascade, identification and removal of foreign substances, activation of the adaptive immune response, and the utilization of physical and chemical barriers against infectious agents.^53^ The vitamin D receptor (VDR) is expressed both in the keratinocytes^54,55^ and cells of the innate immune system such as macrophages and monocytes,^56–59^ thus ensuring its action on two lines of body defence.

The beneficial effects of vitamin D on the innate immune system were appreciated early on, as it was implemented as a treatment of infections for a period longer than 150 years, including mycobacterial diseases, such as tuberculosis and leprosy.^60–63^ Thus, in 1849, Williams reported favourable results after the administration of cod liver oil, an excellent source of vitamin D, in the treatment of patients with tuberculosis.^64^ Half a century later, Niels Finsen successfully used UV light, an effective method to increase vitamin D levels, for the treatment of lupus vulgaris, a form of skin sarcoidosis- receiving the third Nobel prize in Medicine.^6,65^ Moreover, Alfred Windaus, contributed to the discovery of the chemical structure of vitamin D_2_ and vitamin D_3_ found in cod-liver-oil, also receiving the Nobel prize.^7,8,66^ Thereafter, several groups used vitamin D_2_ and D_3_ as a treatment for tuberculosis.^7,67^ Rook et al.^68^ demonstrated in the 1980s that 1,25(OH)_2_D_3_ inhibited the proliferation of M. tuberculosis in cell cultures. Vitamin D enhances the production of defensin β2 and cathelicidin in response to infection by macrophages, monocytes, and keratinocytes.^49^ Humans have only one cathelicidin,^69^ which is produced by cells of the immune system, including neutrophils, macrophages, and cells lining epithelial surfaces that are constantly exposed to potential pathogens such as the skin, the respiratory, and the gastrointestinal tract.^70–72^ Cathelicidin has broad antimicrobial activity against gram-positive and gram-negative bacteria, an effect mediated through cell lysis via cell membrane destabilization,^73^ as well as activity against certain viruses and fungi.^74^ Treatment with 1,25(OH)_2_D_3_ upregulates cathelicidin mRNA in several cell lines, ensuring antimicrobial peptide production on a variety of different cells.^75^ 25(OH)D_3_ is the major circulating form of vitamin D used to determine vitamin D status and is important for local production of 1,25(OH)_2_D_3_, which upregulates cathelicidin production in both skin and macrophages. Exposing human monocytes to pathogens, increases the expression of both 1,25(OH)_2_D_3_ and VDR, thus increasing both the local production of 1,25(OH)_2_D_3_ and the ability of the cell to respond to it.^49^ As keratinocytes possess 25-α-hydroxylase, UV light may directly stimulate cathelicidin production by providing the substrate 25(OH)D_3_ directly from vitamin D3 produced within the skin.^76,77^ Macrophages are phagocytic antigen-presenting cells, which are involved in the first line of defence against pathogens. 1,25(OH)_2_D_3_ has various roles in macrophage differentiation and activation. Macrophage exposure to 1,25(OH)_2_D_3_ can enhance the differentiation of macrophages from monocytes and upon exposure to inflammatory immune signals the expression of 1a-hydroxylase is enhanced, thus allowing the macrophage to locally produce the bioactive metabolite of vitamin D, namely 1,25(OH)_2_D_3_,^42,78^ which is necessary for immune modulation. Macrophages respond to vitamin D increasing their antimicrobial activity in an heterogeneous manner; thus, those activated after an interleukin-15 stimulus respond adequately, in contrast, interleukin-10 stimulus leads to weak responses.^79,80^ Taken together, the ability of the immune cells to hydroxylate 25(OH)D_3_ locally, suggests that in patients with infections it may be better to administer 25(OH)D_3_ rather than hydroxylated metabolites to allow for local production and the feedback system to function.

Neutrophils are the most abundant white blood cell population in the human, and they contribute to a line of defence against microbial pathogens. Neutrophils can clear microbes through many mechanisms including phagocytosis and generation of reactive oxygen species and express a functional vitamin D receptor.^38^ In accordance, 1,25(OH)_2_D_3_ administration has been shown to reduce the production of inflammatory cytokines and reactive oxygen species^81^ and to downregulate neutrophil function and activity.

Monocytes and in particular dendritic cells represent antigen presenting cells, which are important in the initiation of the adaptive immune response. Both cell types can be either immunogenic or tolerogenic and thereby modulate T cell responses.^82,83^ Tolerogenic antigen presenting cells are characterised by a reduced expression of co-stimulatory molecules and a cytokine production favouring regulatory T cell (Treg) induction.^84^ Dendritic cells are antigen presenting cells, which survey the microenvironment and are specialised in antigen uptake and processing. Dendritic cells are crucial regulators of the delicate balance between immunogenicity and immune tolerance.^85^ In dendritic cells 1,25(OH)_2_D_3_ can interfere with the differentiation and maturation process, thus resulting in an altered morphology, phenotype and function leading to a semimature or tolerogenic phenotype.^86,87^ Vitamin D has been shown to manipulate monocytes and dendritic cells at different levels enabling them to exert tolerogenic activities, which could be exploited to better control autoimmune diseases.^86^

## VITAMIN D AND ADAPTIVE IMMUNITY

Although primarily an activator of the innate immune system to enhance immediate response to infection, vitamin D also acts to regulate the adaptive immune system. The adaptive immune system includes both humoral immunity components and cell mediated immunity components, both directed against invading pathogens. Adaptive immunity leads to immunological memory after an initial response to a specific pathogen, resulting in an enhanced response to future encounters with that pathogen^88^ through faster and enhanced production of neutralising antibodies.^89^

Treg cells (Tregs) are an immunosuppressive subpopulation of T cells, which modulate the immune system, maintaining self-tolerance, and preventing autoimmunity.^90^ Vitamin D can promote development and function of Tregs in vitro.^91^ Effector T cells are directly and indirectly affected leading to a shift in the Th1/Th2 balance toward Th2 and a reduction of the Th17 response.^91^ Once T cells are activated, 1,25(OH)_2_D_3_ inhibits IL-2 production.^92^ T cells harbour the vitamin D receptor.^36^ The behaviour of T cells is modulated by vitamin D indirectly via its effects on dendritic cells. The vitamin D receptor is expressed at low levels in freshly isolated CD8+ and CD4+ T cells.^36,40,93,94^ Following activation and addition of 1,25(OH)_2_D_3_ the expression of the vitamin D receptor is induced. In addition, activated CD8+ cells can produce 1a-hydroxylase, which can convert 25(OH)D_3_ to the active 1,25(OH)2D_3_.^95^ Thus, the regulation of T cells responsiveness to vitamin D is a late event.^96^ Vitamin D and 1,25(OH)2D_3_ inhibit T cell proliferation and cytokine production, an event occurring after activation.^36,93^ It has been hypothesised that following an infection, T cells are induced which are important for clearing the pathogen. The effect of vitamin D does not occur until after the T cell response to the infectious organism has begun. In the infection models, T cells eliminate the pathogen, and the antigen is removed from the system, whereas in an immune mediated disease the antigen persists and T cells are chronically activated, producing inflammatory cytokines.^97^ It has been proposed that vitamin D deficiency results in a reduced capacity to turn off T cells following activation.^96^ In a previous study, peripheral blood mononuclear cells which were stimulated with T-cell specific mitogens in the presence of 1,25(OH)_2_D_3_ proliferated less and produced less inflammatory cytokines, including interferon-γ.^98^

B cells express immunoglobulin receptors in their plasma membrane, recognising antigenic epitopes. They produce autoantibodies and form B cell follicles with germinal centre activity. Once activated, B cells can upregulate the expression of vitamin D receptor and 1a-hydroxylase.^99^ 1,25(OH)_2_D_3_ in B cells can induce apoptosis, inhibiting memory B cell formation and preventing differentiation of B cells to immunoglobulin-producing plasma cells.^100^

## VITAMIN D AND AUTOIMMUNITY

Vitamin D has immunomodulatory properties,^50,101,102^ and early on after its discovery, it was shown to have immunostimulatory effects as well.^7^ In the course of the years, and as the autoimmune diseases were found to increase in prevalence,^103^ a worldwide prevalence of vitamin D deficiency was observed,^1,104^ implying a significant role of vitamin D in inducing immune tolerance,^11,12,86^ (**Figure 1**) and a potential role of vitamin D deficiency in the development of autoimmune diseases.^10,105,106^ Extensive research provided evidence that vitamin D deficiency may induce the development of rheumatoid arthritis^13–16,107–109^ and that it is related to its activity and severity^14,16^ (**Table 1**). A cross-talk between oestrogen and vitamin D has been postulated, suggesting a sex-specific effect of vitamin D in autoimmunity.^110^ Research also provided evidence that vitamin D deficiency may be related to systemic lupus erythematosus^18–20 ,22,23^ and multiple sclerosis.^25,27,111–113^ Vitamin D deficiency appears to be also highly prevalent in patients with inflammatory bowel disease^17^ (Crohn’s disease and ulcerative colitis) in relation to disease activity.^114^ Vitamin D supports the integrity of the intestinal barrier and is related to microbiota homeostasis in this cohort of patients^115,116^ and may contribute to the prevention of inflammatory bowel disease by supporting the integrity of the intestinal barrier, ensuring bacterial homeostasis and ameliorating disease progression via anti-inflammatory action.^117^ Vitamin D deficiency in inflammatory bowel disease is aggravated by decreased absorption of the vitamin via the gastrointestinal tract.^116^ Additionally, vitamin D seemed to induce remission in a cohort of patients with Crohn’s disease.^118^ It has been postulated that vitamin D resistance may be observed in some patients necessitating an individualised approach in the treatment of vitamin D deficiency.^119^

**Table 1.**
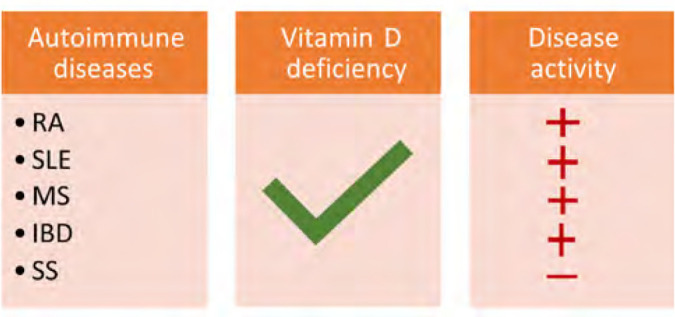
Autoimmune diseases and relationship of disease activity or severity to vitamin D deficiency (RA,^13–16, 21^ SLE,^17–21^ multiple sclerosis,^23–26,98,99^ inflammatory bowel disease,^27,98,99,103–15^ systemic sclerosis^28^)

## CONCLUSION

In conclusion, vitamin D is a likely immunomodulatory agent. It has immune stimulating properties, as it enhances the function of the innate immune system, and it may induce immune tolerance. Vitamin D deficiency may be related to the development of autoimmune diseases.
